# Defining a panel of principal bacteria associated with endometritis

**DOI:** 10.5935/1518-0557.20240088

**Published:** 2025

**Authors:** Délis de Oliveira Ferreira, Martha Giovanna de Macêdo Costa, Raíssa dos Santos Belarmino, Danielle Barbosa Morais, Kyvia Bezerra Mota, Márcio Ferrari, Daniel Carlos Ferreira Lanza

**Affiliations:** 1 Graduate Program in Drug Development and Technological Innovation, Federal University of Rio Grande do Norte, Natal, RN, Brazil; 2 Laboratory of Applied Molecular Biology - LAPLIC, Department of Biochemistry, Federal University of Rio Grande do Norte, Natal, RN, Brazil; 3 Department of Morphology, Federal University of Rio Grande do Norte, Natal, RN, Brazil; 4 Health Care Directorate, Federal University of Rio Grande do Norte, Natal, RN, Brazil

**Keywords:** endometrial dysbiosis, endometrium, bacterial, IVF, fertilization

## Abstract

The aim of this study is to present a panel that includes the main bacterial genera associated with endometritis. We conducted a search using the terms “endometritis women” OR “female endometritis” OR “pelvic inflammatory disease” AND bacteria* OR “uterine microbiome” in two databases: PubMed and Web of Science, without language or publication year restrictions. The panel is based on an analysis of 40 studies published over the past 38 years. We identified 31 bacterial genera, with the following five being the most frequently cited: Chlamydia and Ureaplasma with 11.03% each, Streptococcus and Mycoplasma with 9.56% each, and Enterococcus with 8.09%. Regarding its etiological aspects, we found that bacterial infection is the most prevalent cause of the disease, occurring because of invasive procedures such as curettage, cesarean section, or insertion of intrauterine devices (IUDs), among others. These events facilitate the entry of pathogenic microorganisms into the uterus, resulting in an inflammatory response and subsequent development of endometritis. The main techniques used to detect these pathogens were microbial culture, Polymerase Chain Reaction (PCR), and Next-Generation Sequencing, with microbial culture being the most employed, followed by PCR or a combination of both techniques. This diversity of techniques has significantly expanded our understanding of the presence and identification of microorganisms associated with the pathophysiology of endometritis. Therefore, it is understood that these findings serve as a foundation for further investigations of microorganisms related to endometritis, and such analyses will help to clarify the relationship between endometritis and the bacteria that cause it..

## INTRODUCTION

Endometritis is characterized by a continuous inflammatory process of the endometrium and is histopathologically subdivided into acute and chronic categories (Moreno & Simon, 2018; Kitaya *et al*., 2018). Acute Endometritis (AE) is characterized by microabscess formation and neutrophil invasion on the endometrial epithelium surface due to the presence of aerobic and anaerobic bacteria (Singh & Sethi, 2022). Chronic Endometritis (CE) is marked by plasma cell infiltration in the endometrial stroma, causing prolonged inflammation. CE is also commonly caused by bacterial infection (Kitaya & Yasuo, 2011).

For a long time, it was believed that a healthy uterine cavity was devoid of microorganisms, as cervical mucus was considered an impermeable barrier against ascending bacteria from the vagina (Baker *et al*., 2018; Quayle, 2002). This hypothesis has been refuted by several studies that identified the presence of microorganisms in the uterine cavity of healthy women, noting that the mucus plug incompletely blocks the ascent of bacteria from the vagina and that uterine peristaltic movement assists in translocating particles from the vagina to the uterus (Heinonen *et al*., 1985; Kunz *et al*., 1997; Hansen *et al*., 2014; Chen *et al*., 2017).

In recent years, some studies have indicated that CE might be associated with an altered endometrial microbiome, potentially influencing the implantation process and pregnancy success (Moreno *et al*., 2018; Liu *et al*., 2019). CE is common in patients with unexplained infertility, and when diagnosed and treated, pregnancy rates are higher (Cicinelli *et al*., 2018; Ravel *et al*., 2021; Singh & Sethi, 2022). Kushnir *et al*. (2016) reported that about 46% of infertile patients had CE, especially those with recurrent implantation failure. However, the causal relationship between CE and reproductive failure has not been clearly established to date (Lozano *et al*., 2021).

The prevalence of CE is often underestimated, primarily due to diagnostic difficulties, with approximately 25% of patients showing no symptoms (Singh & Sethi, 2022; Kitaya *et al*., 2018). Kimura *et al*. (2019) report that the prevalence of CE varies from 8% to 72% in the reproductive-age women population, suggesting that this wide variation may be associated with different diagnostic criteria applied to the disease.

Given the current absence of a definitive understanding of the microbiota associated with endometritis, our objective was to formulate an inaugural panel comprising the key bacterial genera implicated in this inflammation. This carefully curated panel is poised to be instrumental in associative studies, aimed at elucidating the intricate relationship between endometritis and its bacterial provocateurs.

## METHODS

We initially identified all studies published up to December 19, 2023, available in the PubMed and Web of Science databases, without language restriction. The search was conducted using the following parameters: endometritis women OR endometritis female OR “Pelvic Inflammatory Disease” AND bacteria* OR uterine microbiome.

To identify the main bacteria associated with endometritis, all titles, abstracts, and full texts of articles without publication date restriction were individually examined. Meta-analyses, and other publications that did not report original clinical data and/or did not have the term “endometritis” in the title or abstract were excluded, as were studies conducted on animal models, book chapters, and articles with unavailable full texts.

Once the main bacteria related to endometritis were identified, the search was expanded to include other already published articles about these target bacteria, as well as review studies to deepen the discussion of this study.

## RESULTS

**Figure 1:**
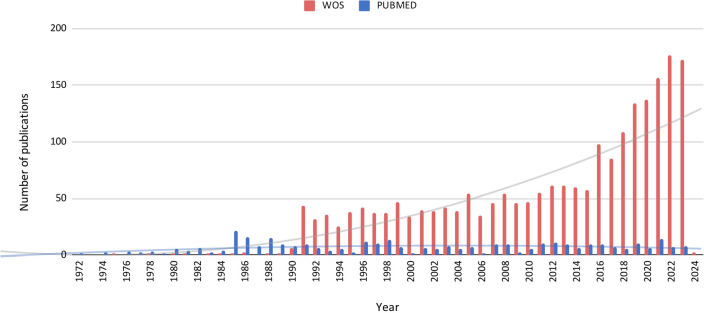
Publication Trends of Articles Mentioning ‘Endometritis’ (1972-2023). This graph depicts the number of articles citing ‘endometritis’ each year, based on data from Web of Science (WOS) and PubMed. The X-axis shows the year of publication, while the Y-axis represents the total number of articles published annually.

We identified 2,539 articles, with 346 in PubMed and 2,193 in Web of Science. Considering the number of publications from 1972, we observed that the number of studies citing the term endometritis increased annually over time, becoming more evident from the 90s (Figure 1).

**Figure 2:**
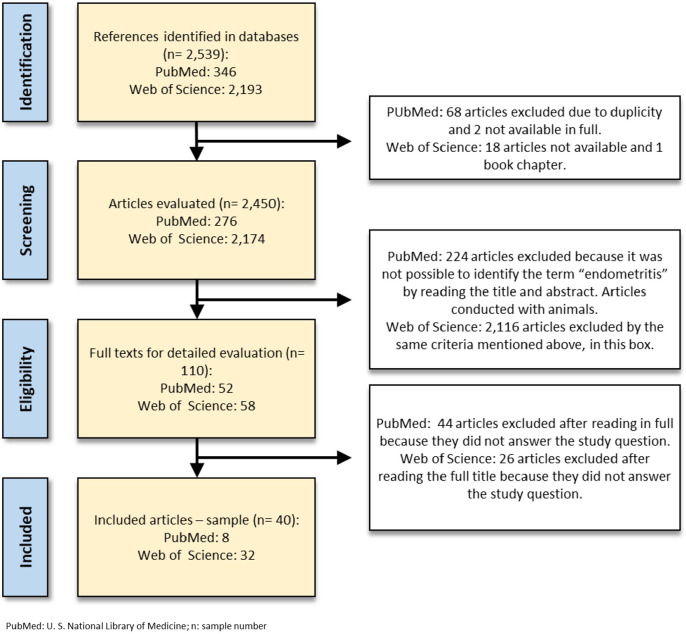
Literature Review and Article Selection Process Flowchart. This flowchart illustrates the methodology followed for the literature review and the criteria for selecting articles for this study

After screening by title and abstract, 68 articles were duplicates, 20 articles unavailable, 1 book chapter, and 2,340 articles that did not present the term “endometritis” or were conducted on other animals, were excluded for not meeting the inclusion criteria. After analyzing 110 articles in full, 70 of them were excluded for not being directly related to the topic. Thus, we considered only studies with women, describing the microbiota related to CE focusing on bacteria, including 40 articles of the initial total (~1.8%) published between 1985 and 2023. The detailed flowchart we used for selecting the articles is presented in Figure 2.

Most studies were conducted in the United States (19 studies), but we also identified works from Finland and Italy with 3 studies each; Israel, Switzerland, Japan, and China with 2 studies each; Germany, Puerto Rico, Turkey, Belgium, Egypt, Kuwait, Spain, and Wales with one study each. Among the age ranges of the patients who participated in the studies, those aged between 20 and 30 years were the most represented (~28% of the works), but the set of works also included studies with younger patients (from 15 years) or older patients (40 to 69 years).

Among all the evaluated works, 16 used microbial culture techniques to investigate the presence of microorganisms related to the condition under study, 9 studies used PCR techniques to identify specific genetic material from relevant bacteria, and 4 articles combined both techniques, leveraging the strengths of each. Notably, only three studies evaluated the endometrial microbiota through next-generation sequencing, including the 16S ribosomal RNA sequencing approach. The details about each of the studies analyzed also considering the main outcomes related are available in Table 1.

**Table 1:** Main characteristics of the works selected for this study.

Year	Country	Bacteria	Detection methods	Outcome	Reference
1985	USA	*Escherichia coli, Klebsiella pneumoniae, Proteus* *mirabilis, Enterobacter sp. and Pseudomonas sp.*	Microbiological culture medium	Postpartum endometritis	Gibbs *et al*. (1985)
1985	Finland	*Chlamydia trachomatis, Neisseria gonorrhoeae and Streptococcus* *agalactiae*	Microbiological culture medium	Salpingitis	Paavonen *et al*. (1985)
1986	USA	*Streptococcus agalactiae,* *Streptococcus sp.,* *Enterococcus sp., Gardnerella vaginalis, Escherichia* *coli, Enterobacteriaceae, Bacteroides bivius,* *Peptoniphilus asaccharolyticus,* *Peptostreptococcus sp.,* *Peptococcus sp., Bacteroides fragilis, Ureaplasma urealyticum,* *Mycoplasma hominis, Chlamydia trachomatis, Streptococcus sanguis, Streptococcus* *constellatus, Streptococcus morbillorum and Staphylococcus* *aureus*		Postpartum fever	Rosene *et al*. (1986)
1986	USA	*Staphylococcus sp.,* *Lactobacillus sp., Streptococcus sp., Streptococcus sp., Enterobacteriaceae,* *Eubacterium sp., Peptococcus sp., Peptostreptococcus* *sp., Clostridium botulinum,* *Bacteroides sp., Fusobacterium sp. and Ureaplasma urealyticum*	Microbiological culture medium	Longer hospital stay	Williams *et al*. (1987)
1990	USA	*Clamydia trachomatis,* *Mycoplasma sp., ureaplasma urealyticum, Enterococcus* *faecalis, Bacteroides bivius and* *Bacteroides fragilis*		-	Martens *et al*. (1990)
1991	USA	*Streptococcus agalactiae and Enterococcus faecalis*	Microbiological culture medium, gram stain and gas-liquid chromatography	Postpartum endometritis	Watts *et al*. (1991)
1992	USA	*Escherichia coli, Citrobacter sp.,* *Enterococcus sp., Streptococcus agalactiae, Streptococcus* *pyogenes, Staphylococcus* *aureus, Staphylococcus* *epidermidis, Corynebacterium sp., Proteus mirabilis,* *Enterobacter aerogenes,* *Enterobacter cloacae, Gardnerella vaginalis, Streptococcus viridans,* *Pseudomonas aeruginosa,* *Neisseria gonorrhoe and* *Chlamydia trachomatis*	Microbiological culture medium, gram stain and gas-liquid chromatography	Postpartum endometritis	Apuzzio *et al*. (1992)
1993	Wales	*Prevotella melaninogenica*	Microbiological culture medium	Endometritis, salpingitis, tubo- ovarian and pelvic abscesses	Duerden (1993)
1995	USA	*Ureaplasma urealyticum*	Microbiological culture medium	Postpartum endometritis	Andrews *et al*. (1995)
1996	USA	*Neisseria gonorrhoea, Chlamydia trachomatis and Prevotella bívia*	Microbiological culture medium	Salpingitis	Hillier *et al*. (1996)
1998	USA	*Neisseria gonorrhoea and Chlamydia trachomatis*	Microbiological culture medium	Endometritis in the proliferative phase	Korn *et al*. (1998)
1999	Finland	*Chlamydia trachomatis*	PCR	-	Paukku *et al*. (1999)
1999	Israel	*Mycoplasma sp.*	Microbiological culture medium	Postpartum endometritis	Sherman *et al*. (1999)
1999	Puerto Rico	*Streptococcus agalactiae and Escherichia coli*	Microbiological culture medium	Postpartum endometritis and intra-amniotic infection	Krohn *et al*. (1999)
2001	USA	*Chlamydia trachomatis*	PCR	-	Mount *et al*. (2001)
2002	Finland	*Neisseria gonorrhoeae and Chlamydia trachomatis*	Microbiological culture medium	Severe abdominal pain and embryo implantation failures	Eckert *et al*. (2002)
2002	USA	*Mycoplasma genitalium*	PCR	Mild or moderate abdominal pain	Cohen *et al*. (2002)
2003	Israel	*Ureaplasma urealyticum,* *Enterobacter sp., Streptococcus agalactiae, Staphylococcus sp.,* *Enterococcus sp. and Proteus mirabilis*	Microbiological culture medium	Premature birth	Chaim *et al*. (2003)
2004	USA	*Chlamydia trachomatis, Neisseria gonorrhoeae, Mycoplasma* *hominis, Ureaplasma urealyticum, Gardnerella vaginalis,* *Prevotella bivia, Prevotella disiens, Prevotella buccalis,* *Prevotella buccae, Preveotella sp., Bacteroides ureolyticus,* *Bacteroides merdae, Bacteroides sp., Fusobacterium nucleatum,* *Fusobacterium sp., Veillonella sp.,* *Peptostreptococcus anaerobius,* *Peptoniphilus asaccharolyticus,* *Peptostreptococcus magnus,* *Peptostreptococcus prevottii,* *Peptostreptococcus tetradius,* *Peptococcus niger and Peptostreptococcus sp.*	Microbiological culture medium	Bacterial vaginosis	Haggerty *et al*. (2004)
2006	USA	*Trichomonas vaginalis*	Microbiological culture medium and PCR	Acute endometritis	Cherpes *et al*. (2006)
2006	Germany	*Enterococcus sp. and Peptostreptococcus magnus*	Microbiological culture medium	Xanthogranulomatous endome- tritis	Noack *et al*. (2006)
2008	Switzerland	*Ureaplasma urealyticum,* *Chlamydia trachomatis and Mycoplasma genitalium*	Microbiological culture medium and PCR	-	Cicinelli *et al*. (2008)
2009	Switzerland	*Ureaplasma urealyticum*	Microbiological culture medium and PCR	Endometritis	Cicinelli *et al*. (2009)
2013	USA	*Escherichia coli and Enterococcus sp.*	Blood culture	Endometritis, chorioamnionitis and urosepsis	Cape *et al*. (2013)
2014	Italy	*Mycoplasma sp., Ureaplasma sp. and Chlamydia sp.*	Microbiological culture medium and PCR	Recurrent miscarriage	Cicinelli *et al*. (2014)
2014	Türkiye	*Mycoplasma sp. and Ureaplasma sp.*	MycoView - molecular test PCR-based	Abnormal implantation of the placenta, placent previa and endometritis	Aydogan *et al*. (2014)	
2015	Italy	*Mycoplasma sp. and Ureaplasma urealyticum*	Immunoassay and RT - PCR	Impaired endometrial receptivity and implantation failure	Cicinelli *et al*. (2015)	
2016	USA	*Sneathia sanguinegens,* *Leptotrichia amnionii, Atopobium vaginae,* *Ureaplasma urealyticum,* *Ureaplasma parvum, Chlamydia trachomatis and Neisseria gonorrhoeae*	PCR	Bacterial vaginosis, pelvic inflammatory disease, subsequent infertility	Haggerty *et al*. (2016)	
2017	Belgium	*Leptotrichia amnionii*	PCR	Postpartum endometritis	Masschaele *et al*. (2018)	
2018	Italy	*Enterobacteriaceae, Enterococcus sp., Streptococcus sp.,* *Staphylococcus sp., Mycoplasma sp. and Ureaplasma sp.*	Molecular microbiology	-	Moreno *et al*. (2018)	
2018	USA	*Chlamydia trachomatis e Neisseria gonorrhoeae*	PCR	-	Zheng *et al*. (2018)	
2018	USA	*Mycoplasma genitalium*	PCR	Endometritis and vaginal bacteremia	Taylor *et al*. (2018)	
2018	USA	*Chlamydia trachomatis*	Microbiological culture medium and PCR	Premature birth, premature rupture of membranes, endometritis and intrauterine fetal death	Olson-Chen *et al*. (2018)	
2020	USA	*Trichomonas vaginalis*	Microscopy	Persistent endometritis, infertility, recurrent Pelvic Inflammatory Disease (PID), chronic pelvic pain, lower pregnancy and live birth rates	Wiringa *et al*. (2020)	
2021	Egypt and Kuwait	*Mycoplasmas hominis,* *Ureaplasma sp. , Enterococcus faecalis, Chlamydia trachomatis,* *Escherichia coli, Streptococcus viridans, Streptococcus bovis,* *Staphylococcus Haemolyticus and Candida albicans*	Microbiological culture medium and staining immunohistochemistry	Recurrent miscarriage	Farghali *et al*. (2021)	
2021	Spain	*Ralstonia sp. and Gardnerella sp.*	PCR	-	Lozano *et al*. (2021)	
2021	China (Nanchang)	*Gardnerella sp.*	Sequencing new generation	Implantation failure in IVF patients	Chen *et al*. (2021)	
2022	Japan	*Escherichia coli, Enterococcus faecalis, Streptococcus* *sp. Staphylococcus sp.,* *Mycoplasma, Ureaplasma sp. and* *Mycobacterium sp.*	PCR	-	Kitaya *et al*. (2022)	
2022	Japan	*Streptococcus sp., Enterococcus sp. and Atopobium sp.*	Sequencing new generation	Unexplained infertility	Tanaka *et al*. (2022)	
2023	China	*Staphylococcus, Gardnerella,* *Atopobium, Streptococcus,* *Peptostreptococcus, Chlamydia,* *Fusobacterium, Acinetobacter,* *Lactobacillus uteri, Lactobacillus,* *Gardnerella, Bifidobacterium,* *Streptococcus, Argyromonas,* *Prevotella, Porphyromonas and Escherichia*	Pathological examination and 16S ribosomal RNA sequencing	Infertility, Chronic endometritis and Endometrial polyps	Liang *et al*. (2023)	

**Figure 3:**
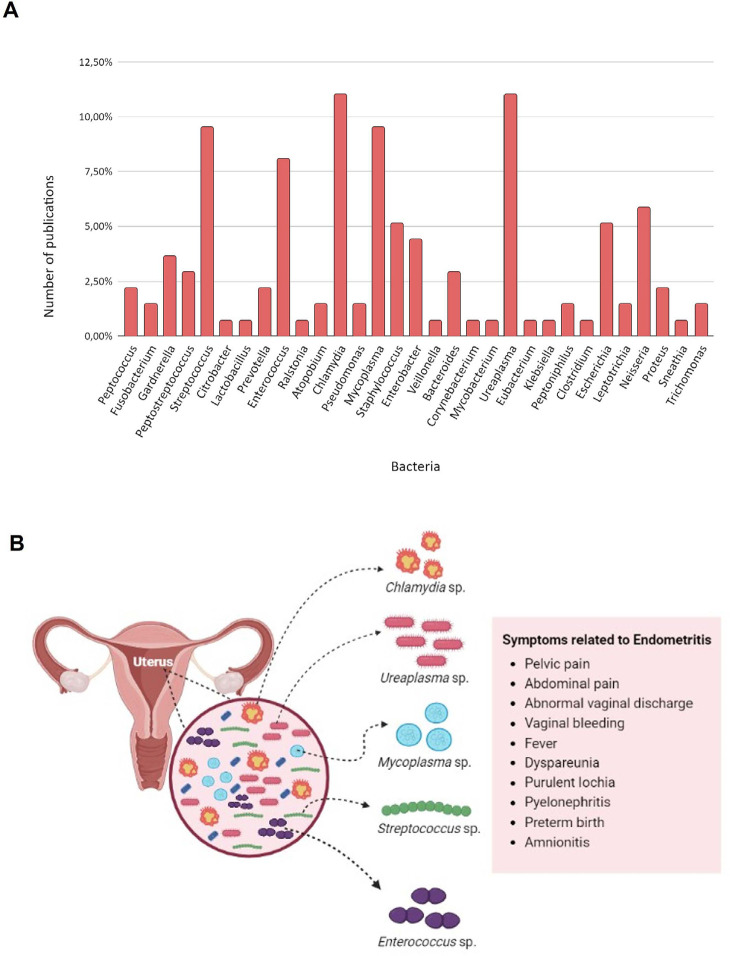
Bacteria and Common Symptoms Associated with Endometritis. (A) Bacteria Linked to Endometritis with the Highest Number of Mentions in Articles Published from 1985 to 2023. (B) Common Symptoms Caused by Dysbiosis Due to the Proliferation of These Bacteria in the Endometrial Tissue.

We identified 75 bacterial genera in the endometrial microbiome of patients with endometritis. Among these, 31 genera associated with the disease were observed in 3 or more articles, with *Streptococcus sp., Enterococcus sp., Chlamydia sp., Mycoplasma sp*., and *Ureaplasma sp*. being the 5 most recurrent genera, all cited in more than 10 works (Figure 3A). Although endometritis has subtle symptomatology, the selected studies highlight the main symptoms associated with the disease: pelvic and abdominal pain, abnormal vaginal discharge, vaginal bleeding, fever, dyspareunia, purulent lochia, pyelonephritis, preterm labor, and amnionitis (Figure 3B).

## DISCUSSION

Bacterial colonization of the endometrium can be considered a key factor in the pathogenesis of endometritis. Our comprehensive analysis underscores that various bacterial genera can reach the endometrium via different routes, with the ascending pathway being the most common, where bacteria present in the vagina or cervix migrate to the uterus. Under these circumstances, during the menstrual period when the cervix is open, the risk of bacterial contamination is believed to be higher. Additionally, invasive medical procedures, such as the insertion of IUDs or dilation, curettage, and delivery methods, can facilitate the entry of bacteria into the endometrium.

As expected, we identified a significant increase in the number of studies related to endometritis over time, with the 1990s marked by a rise in publications mentioning “endometritis.” The selected studies provided important information about the symptoms associated with endometritis, as well as a growing trend in the use of molecular biology techniques to aid in characterizing the endometrial microbiome.

The collective analysis of the 40 studies selected in this research presents the most comprehensive bacterial profile of endometritis available to date, encompassing 75 genera associated with the disease. Of these, 31 genera have been frequently cited in publications over the past 38 years (Figure 3). Among them, *Chlamydia sp., Ureaplasma sp., Mycoplasma sp., Streptococus sp*., and *Enterococcus sp*. can be validated as a reduced panel for initial screenings at a lower cost.

While some authors have pointed to *Gardnerella sp*. and *Neisseria sp*. as the main pathogens associated with endometritis (Singh & Sethi, 2022; Qin & Xiao, 2022), these genera were not the most frequently mentioned in publications mapping the endometrial microbiota in patients with the disease, being cited in 5 and 8 publications, respectively.

In the study conducted by Leoni *et al*. (2019), bacterial genera that make up the endometrial microbiome of healthy patients were identified. Among these discoveries, the presence of *Cutibacterium sp., Escherichia sp., Staphylococcus sp., Acinetobacter sp., Streptococcus sp*., and *Corynebacterium sp*., identified in most samples, stand out. Although these genera are commonly associated with a healthy microbiota, it is intriguing to note that *Escherichia sp., Staphylococcus sp*., and *Streptococcus sp*. also appear associated with endometritis (Table 1). This duality suggests a complex interaction between bacterial composition and endometrial health, opening further avenues for investigation using more modern and comprehensive methodologies, which allow for a more precise and individualized assessment of all bacterial communities present in the sample.

Indeed, we identified only 3 studies that used next-generation sequencing for endometritis evaluation (Chen *et al*., 2021; Tanaka *et al*., 2022; Liang *et al*., 2023). Considering only the study by Liang *et al*. (2023), 5 new bacterial genera were associated with endometritis (*Atopobium sp., Acinetobacter sp., Bifidobacterium sp., Argyromonas sp*., and *Porphyromonas sp*.). This discovery suggests that the complexity of the interaction between the microbiome and endometritis is broader than previously thought and may have crucial implications for understanding the etiology of endometritis, potentially influencing more effective diagnostic and therapeutic strategies. The absence of mention of these genera in the last 38 years highlights the importance of continuous and updated research in the field of microbiology and reproductive health.

Based on our revision, we believe it is possible to establish a relationship between acute endometritis and chronic endometritis. Although the two subtypes of the disease have distinct microbial profiles, infections caused by *Neisseria gonorrhoeae* or *Chlamydia trachomatis* may initially be associated with acute endometritis but can persist in a chronic endometritis scenario, especially within the context of pelvic inflammatory disease (Kitaya *et al*., 2018; Singh & Sethi, 2022). In these cases, the progression of the bacterial infection may result in chronic inflammation of the endometrium, characteristic of chronic endometritis. However, this association still requires further validation, and we believe that the foundation provided here may support future studies in this area.

Given that this work also intends to establish an association between clinical practice and endometrial microbiota, we have listed a set of primary symptoms, possibly resulting from the local inflammatory response, which can be used as a baseline criterion for anamnesis. These are: pelvic and abdominal pain, abnormal vaginal discharge, vaginal bleeding, fever, dyspareunia, purulent lochia, pyelonephritis, premature birth, and amnionitis (Figure 3B). We believe that the application of this panel of symptoms, in conjunction with the bacterial genera panel presented here, can be used for associative studies, allowing for standardization in the diagnosis and indication of treatments for endometritis.

## CONCLUSION

Our study offers insight into the intricate relationship between endometritis, its bacterial causatives, and consequent clinical impacts. By unveiling 31 bacterial genera, this research paves the way for an innovative diagnostic framework for endometritis, aiding in clinical diagnosis since this disease is usually asymptomatic and related to adverse outcomes in female fertility. A specialized subset, comprising *Chlamydia sp., Ureaplasma sp., Mycoplasma sp., Streptococcus sp*., and *Enterococcus sp*., stands out for cost-effective preliminary screening. These findings underscore the importance of identifying and monitoring these bacteria for a more effective approach in treating endometritis, while also providing a valuable foundation for future research aimed at developing more targeted and efficient therapies.
